# The Pampa del Indio Project: long-term variations of *Trypanosoma cruzi* infection in *Triatoma sordida*, a non-target invasive vector

**DOI:** 10.3389/fpara.2026.1953889

**Published:** 2026-10-12

**Authors:** M. V. Cardinal, S. Piñero, G. F. Enriquez, M. S. Gaspe, N. P. Macchiaverna, A. Alvedro, R. E. Gürtler

**Affiliations:** 1 Laboratorio de Eco-Epidemiología, Departamento de Ecología, Genética y Evolución, Facultad de Ciencias Exactas y Naturales Universidad de Buenos Aires, Buenos Aires, Argentina; 2 Instituto de Ecología, Genética y Evolución de Buenos Aires (IEGEBA), Consejo Nacional de Investigaciones Científicas y Técnicas (CONICET)-Universidad de Buenos Aires, Buenos Aires, Argentina; 3 Departamento Agronomía y Ambiente, Facultad de Ingeniería y Ciencias Exactas, Universidad Argentina de la Empresa (UADE), Buenos Aires, Argentina

**Keywords:** Argentina, chagas disease, gran chaco, secondary vectors, *Triatoma sordida*

## Abstract

**Introduction:**

The Gran Chaco region still faces vector-borne transmission of *Trypanosoma cruzi* by the main vector, *Triatoma infestans*. In Pampa del Indio, a municipality belonging to the Argentinian Chaco, a long-term research and intervention program achieved the quasielimination of *T. infestans* from rural houses and halted the domestic vector-borne transmission of *T. cruzi*. Here, we evaluate the occurrence and distribution of *T. cruzi* infection in *Triatoma sordida sensu lato*, a secondary species that frequently invades rural domiciles and colonizes the peridomestic environment, over 2007-2016. Based on the prevalence of infection reported across the Gran Chaco region in the absence of *T. infestans* and given the limited sensitivity of optical microscopy, we hypothesized that *T. cruzi* infection in *T. sordida* post-intervention would be higher than in our previous reports (0-6%).

**Methods and results:**

Of 10,728 *T. sordida* collected at 528 premises along the intervention, we examined 1,946 specimens by OM or vkDNA-PCR and found a naïve prevalence of *T. cruzi* infection of 1.44% (95% CI: 0.10% –2.07%). The mean number of infected *T. sordida* per house was 1.12 (SD = 0.33). Infection was higher in adults rather than nymphs; maximal in domiciles, intermediate in peridomestic kitchens, storerooms and granaries, and minimal in other peridomestic sites. Spatial analysis identified an infection hotspot in the northwestern section of the municipality. By means of single-season occupancy models, we estimated that the detection probability of *T. cruzi* infection was method-dependent, with OM detecting at most 73% of the truly infected *T. sordida* (assuming 100% detectability with vkDNA-PCR). Across nearly a decade of vector surveillance and control, *T. cruzi* infection in *T. sordida* remained <2%, and ≤5% of the houses harboring *T. sordida* exhibited infected specimens.

**Discussion:**

We report relatively low levels of *T. cruzi* infection, one component of Chagas disease transmission risk, in the currently most abundant triatomine vector in Pampa del Indio. Given the enzootic nature of *T. cruzi* transmission cycles, our imperfect knowledge on how they function, and the finding of infected *T. sordida* throughout the post-intervention phase, sustained surveillance within an adaptive management scheme is needed to guarantee long-term vector-borne transmission control.

## 1 Introduction

Chagas disease, caused by the protozoan *Trypanosoma cruzi* (Kinetoplastida), is mainly transmitted to humans by the vectorial route and vertical transmission (World Health Organization, 2026). Vector-borne transmission is mediated by triatomines, a diverse group of obligate hematophagous insects (Reduviidae, Triatominae) (Weirauch, 2021). Triatomine vectorial capacity depends on important biological traits that differ among species, like anthropophilic blood-feeding, domiciliation habits, bloodmeal size, feeding and defecation rates, and parasite load in feces (Gürtler et al., 2021). Along the American continent, five species have been recognized as the most important from the epidemiological point of view: *Triatoma infestans*, *Panstrongylus megistus*, *Triatoma dimidiata*, *Rhodnius prolixus* and *Triatoma brasiliensis* (Guhl, 2017). Consequently, they have been the target of vector-control campaigns that over the decades have drastically reduced their distribution ranges and human incident cases (Pan American Health Organization, 2026).

The role of secondary vectors (domiciliary or intrusive triatomines, following Noireau and Dujardin (2010) in the epidemiology of *T. cruzi* has been the matter of intense research. There is strong evidence that some species can maintain a sylvatic transmission cycle with high levels of *T. cruzi* infection, like *Mepraia spinolai* associated with the rodent *Octogon degus* in Chile (Botto-Mahan et al., 2005), and *Triatoma eratyruciforme* associated with *Microcavia australis* in the Calchaquíes valleys in Argentina (Cecere et al., 2016). In Texas, opportunistic feeding habits and high *T. cruzi* prevalence were reported in native triatomine species like *Triatoma sanguisuga* and *Triatoma gerstaeckeri* (Balasubramanian et al., 2022). Where secondary vectors invade domiciles, their abundance, feeding patterns, infection prevalence, and the degree of overlapping between domestic and sylvatic transmission cycles are key features to assess transmission risk to humans by these species.

Hand in hand with the control of the main triatomine vector, secondary species have emerged and increased their occurrence in (peri)domestic habitats. This is the case of *Triatoma brasiliensis* in Brazil and *Triatoma sordida* in Brazil and the Gran Chaco region in South America. In Minas Gerais, Brazil, *T. sordida* predominated as early as the 1990s (Diotaiuti et al., 1993) as the elimination of *T. infestans* from domestic and peridomestic ecotopes proceeded. Frequent finding of domestic infestations with *T. sordida* was also reported in Bolivia (Brenière et al., 1998), Paraguay (Sánchez et al., 2016, 2020), and house invasion by *T. sordida, T. garciabesi* and *T. guasayana* adults were reported across Argentina (Bar et al., 1993; Cardozo et al., 2021; Gürtler et al., 1999; Rodríguez-Planes et al., 2018; Wisnivesky-Colli et al., 1993).

Comparison among study areas and further generalizations are hampered by the fact that *T. sordida* belongs to the Sordida subcomplex (Triatominae) comprising at least six species, *Triatoma garciabesi*, *Triatoma jurbergi*, *Triatoma matogrossensi*, *Triatoma vandae*, *Triatoma rosai* and *T. sordida* (Burgueño-Rodríguez et al., 2025). The latter has been divided in two species based on cytogenetics, molecular data and morphometry: *T. sordida* from Argentina [named as *T. rosai* (Alevi et al., 2020a)] and *T. sordida* from Brazil, Bolivia and Paraguay (Nattero et al., 2017). The inclusion or exclusion of *T. guasayana* and *T. patagonica* within the *T. sordida* subcomplex depends on markers and/or criteria employed (reviewed in Burgueño-Rodríguez et al., 2025). In this manuscript, we will refer to *T. sordida sensu lato* to allow a broad comparison with previous studies in which these species were not differentiated other than by morphological aspects.

One key component of transmission risk is the prevalence of *T. cruzi* infection in the vectors. In *T. sordida*, it ranged from 0.5-16.2% in Brazil (Alevi et al., 2020b), and reached 16-21% in Bolivia (Brenière et al., 1998; Noireau et al., 1997) and Paraguay (21% overall, range: 16-46%) (Sánchez et al., 2020). In Argentina, very low levels of *T. cruzi* infection (<1%) were reported in peridomestic *T. garciabesi* and *T. guasayana* during five years of selective house spraying with pyrethroid insecticides in Santiago del Estero (Cecere et al., 1999). In Pampa del Indio (Chaco Province), *T. cruzi* prevalence in *T. sordida* was very low (0-1%) both at baseline and six months after a community-wide residual spraying of rural houses when the insects were examined by optical microscopy (Macchiaverna et al., 2015). However, when tested by vkDNA-PCR, the prevalence of infection reached 6% on both occasions (Macchiaverna et al., 2015), revealing underestimated *T. cruzi* infections by optical microscopy as reported in Brazilian *T. sordida* and other triatomines (Minuzzi-Souza et al., 2018) and reviewed recently (Cardinal et al., 2025). In light of these results, previous reports of low levels of *T. cruzi* infection (≤1%) in *T. sordida* across Argentina have underestimated the true prevalence of infection.

Bloodmeal sources, particularly whether *T. sordida* feeds on mammals or birds (which are refractory to *T. cruzi* infection) and how often, may also explain the wide variations of *T. cruzi* infection reported. In Brazil, the main bloodmeal sources for sylvatic *T. sordida* were opossums and rats in hollow trees and bark, and birds in different species of palm trees (Diotaiuti et al., 1993). Nevertheless, a recent study from northern Minas Gerais (Brazil), encompassing >1,400 peridomestic *T. sordida*, reported chickens as the main bloodmeal source and relatively low parasite burdens, which may also contribute to imperfect detection of infection by classical parasitological methods (Valença-Barbosa et al., 2025). Humans, marsupials, rodents and chickens were frequent bloodmeal sources in *T. sordida* from Paraguay (Sánchez et al., 2018, 2020), and chickens and turkeys in Argentina (Macchiaverna et al., 2015), where the *T. cruzi* genotypes infecting peridomestic *T. sordida* were linked to local *Didelphis albiventris* opossums (Macchiaverna et al., 2025).

As part of a long-term research and control program on Chagas disease across Pampa del Indio municipality (Chaco province, northeastern Argentina), *T. infestans* was nearly eliminated from rural households and vector infection declined concomitantly (Gürtler et al., 2023a, b). Sustained control actions led to the apparent interruption of domestic vector-borne *T. cruzi* transmission to humans (Cardinal et al., 2021). *Triatoma infestans* was the target of chemical control, and transient findings of *T. sordida* adults in domiciles did not qualify for focal control across the surveillance phase. Here, we evaluate the occurrence and distribution of *T. cruzi* infection in *T. sordida* along the intervention program (2007-2016). Based on the prevalence of infection reported across the Gran Chaco region in the absence of *T. infestans* and given the limited sensitivity of optical microscopy, we hypothesized that *T. cruzi* infection in *T. sordida* post-intervention would be higher than reported previously (0-6%) (Macchiaverna et al., 2015; Gaspe et al., 2018).

## 2 Materials and methods

### 2.1 Study area

Rural sections of Pampa del Indio municipality (approximately 1800 km^2^) encompassed 1,546 housing units at baseline; and were divided into four areas (Area I-IV) for operational reasons. The rural villages were inhabited by Indigenous Qom and Creoles families in similar proportions, though the former predominated in Areas II and III; both population subgroups exhibited high levels of social vulnerability and unmet basic needs. The study areas were described in detail elsewhere (Gurevitz et al., 2011; Provecho et al., 2017; Gaspe et al., 2015; Macchiaverna et al., 2024).

The research program was launched in 2007 and consisted in successive phases (Gürtler et al., 2023a). Firstly, a district-wide exploratory survey was conducted in 2007. Secondly, a baseline census of all rural houses, entomological assessment of triatomine infestation by timed manual collections, and an environmental and sociodemographic questionnaire was conducted in every house. Immediately after, the attack phase took place; it consisted in an area-wide spraying with pyrethroid insecticides regardless of the infestation status of each rural house. Triatomine searches and insecticide spraying were performed by vector control personnel from the National or Chaco Province Chagas disease programs, and supervised by a member of the research team. Activities began in Area 1 and were scaled up to cover the remaining operational areas. After the massive spraying a surveillance phase followed. For this, we promoted community participation and asked householders to notify the occurrence of infestation by *T. infestans* and to collect any triatomine arriving to their homes. Community-based surveillance was combined by research-team led periodic surveys of house infestation. Whenever a house was found infested by *T. infestans*, the house compound was sprayed with insecticide. The occurrence of *T. sordida* did not qualify for focal control unless domestic colonies were detected. Overall, 92.7% of the registered house units were sprayed during the attack phase, and a mean of 0.13 insecticide sprays per house took place during the surveillance phase (SD = 0.44) (Gürtler et al., 2023a). We divided the surveillance phase into an early intervention period spanning 4–28 MPI (when malathion was selectively applied to extinguish the persistent foci of *T. infestans*), and a late intervention period spanning 34–100 MPI.

### 2.2 Triatomine collection

We collected triatomines using three methods: timed-manual searches (the standard way of assessing house infestation); householder collection of any triatomine they sighted (and kept into the provided plastic bags), and insecticide spray-related triatomine collections (Gürtler et al., 2023a). Collected triatomines were kept in labeled plastic bags according to collection site by house, and then taken to the field laboratory where they were identified to species following Lent and Wygodzinsky (1979), and counted by sex and stage. Herein, the study specimens identified as *T. sordida* are considered as *T. sordida sensu lato*.

### 2.3 Diagnosis of *T. cruzi* infection

We examined live third-instar nymphs and later stages by direct microscopy at 400× within 20 days of collection prioritizing *Triatoma infestans* over *T. sordida* specimens and those collected by timed manual searches over insects collected by other methods (Gürtler et al., 2023b). Optic microscopy examination mainly concentrated in insects collected in Area I during the first three years of the intervention program; examination effort varied widely among study areas and surveys. All insects were preserved at −20°C. DNA extraction and molecular diagnosis of *T. cruzi* infection was performed as described (Cardinal et al., 2025). We considered an insect infected with *T. cruzi* when it was OM-positive or vkDNA-PCR-positive. We identified *T. cruzi* Discrete Typing Units (DTUs) in the positive insects following the PCR-based protocol described by Burgos et al. (2007). Brieflly, we targeted three different genomic markers: the intergenic region of spliced leader genes (SL-IR), the D7 domain of the 24Sα ribosomal RNA genes, and the genomic marker A10. Amplification of the SL-IR used three independent hot-start PCR reactions (SL-IR I, SL-IRac and SL-IR II).

### 2.4 Study design

We compiled in our infection database molecular diagnosis results previously reported (Macchiaverna et al., 2015). We expanded the molecular diagnosis effort with the aim of analyzing *T. sordida* from all infested houses at the last survey (April 2016). We also examined *T. sordida* collected by householders and sampled up to 10 insects per infested house from surveys in which few *T. sordida* had been examined by OM. Most insects that were not examined by optic microscopy at baseline were dead at the moment of examination or had no intestinal contents. Adult bugs and third-, fourth- or fifth-instar nymphs not examined for infection by OM were randomly selected for subsequent diagnosis via vkDNA-PCR.

### 2.5 Data analysis

This study was performed in accordance with the STROBE checklist. The current dataset includes the *T. sordida* specimens previously reported by Maffey et al., 2012 (n = 9), Macchiaverna et al., 2015 (n = 205) and Gaspe et al., 2018 (n = 42). We calculated Agresti-Coull binomial 95% confidence intervals (95% CI) for the percentage of insects examined by different methods or for infection prevalence by developmental stage. Wilson 95% CIs were used when the number of examined bugs was <40 or the proportions were near 0 or 1 (Brown et al., 2001). Confidence intervals and Z-tests to compare proportions of examined insects were calculated using Epitools (Sergeant, 2018). Fisher’s exact test was used to test for differences in prevalence among areas and collection ecotopes.

#### 2.5.1 Accounting for imperfect detection

To account for imperfect detection, a random sample (n = 118) of frozen microscopy-negative *T. sordida* was tested by molecular methods (**Supplementary File 1**). These triatomines were mainly collected at baseline and 6 months post-spraying. OM-positive *T. sordida* were tested by vkDNA-PCR and analyzed for DTU identification (Maffey et al., 2012). We estimated the probability of detecting a *T. cruzi* infection by OM or vkDNA-PCR using single-season occupancy models implemented in the software PRESENCE 2.13.47 (Hines, 2006) and the hierarchical-modeling approach followed in Cardinal et al. (2025). Briefly, the occupancy model creates two linked logit models: one for occupancy probability (Ψ) and one for detection probability (“p”), with Ψ being conditional on p, these models work together by incorporating detection history across multiple observations to jointly estimate Ψ and p. In this case, we have two detections per sample. The detection history is either 00, 01 or 11 (being the results of OM-diagnosis or vkDNA-PCR diagnosis, respectively). The occupancy probability model (Ψ) estimates the probability of infection in a bug based on the chosen covariates (in this case, based on bug stage and collection ecotope). The model first estimates logit coefficients, which are values on the log-odds scale, for each occupancy covariate or detection method relative to a baseline. To obtain interpretable probabilities, these logit values are back-transformed using the inverse logit function. Here, we examined whether the occurrence of infection varied according to bug stage (nymphs versus adults) and collection ecotope (peridomestic sites associated with chickens or poultry versus all other habitats). We constructed five models that expressed different *a priori* hypotheses, where “Ψ” is the occupancy probability (i.e.: probability of being infected with *T. cruzi* or the true prevalence of *T. cruzi* infection) and “p” the detection probability:

Model 1: Ψ (.), p(.): uniform detection and uniform infection probabilities.
Model 2: Ψ (.), p(method): detection probability varying with the method, uniform infection probability.
Model 3: Ψ (adult), p(method): detection probability varying with the method, infection probability varying with triatomine stage.
Model 4: Ψ (ecotope), p(method): detection probability varying with the method, infection probability varying with triatomine collection ecotope.
Model 5: Ψ (adult+ecotope), p(method): method-dependent detection and infection occurrence varying with triatomine stage and collection ecotope.

We denoted within brackets the covariates considered for each probability. The null model, with no covariates for occupancy and uniform detection probability, was denoted “Ψ(.)p(.)”, where “.” denotes no covariate. We employed Akaike’s Information Criterion (AIC) for model selection; hypotheses represented by models with lower AIC values are better supported by the data. Based on the estimated OM detection probability we adjusted the naïve prevalences and calculated weighted overall prevalences taking into account the number of insects examined by OM or by vkDNA-PCR.

#### 2.5.2 Spatial analysis

We performed a global spatial analysis of the occurrence of *T. cruzi* infection by considering houses harboring *T. sordida* specimens examined for infection as a spatial point pattern. We aimed to detect the occurrence of clustering of *T. cruzi* infection anywhere within the study area via the K-function using random labeling as the null hypothesis (Getis and Ord, 1996; Waller and Gotway, 2004). For this we calculated the statistic L, which is the linearized K-function. We used the L(r) function implemented in ‘spatstat’ package (Baddeley and Turner, 2005), and L(r) values and confidence envelopes were computed from the mean of 25 replicates of 999 Monte Carlo simulations. The maximum radius employed was 5,000 m. We used a local spatial statistics, Getis (Gi[d]), to identify epicenters of infection clustering between 0 and 5,000 m by comparing the Gi[d] values at all locations within specified distances (d) of the location under consideration. This statistic is a ratio of local averages around the location (i) over the global average of the variable of interest (in this case, occurrence of infection) for the study area (Fortin and Dale, 2005). For these analyses we employed all the infections detected along the 2007–2016 period and included three houses located in the rural-periurban interface harboring *T. cruzi*-infected and non-infected *T. sordida* (Provecho et al., 2014).

## 3 Results

### 3.1 Triatomine infection

A total of 10,728 specimens of *T. sordida* were collected from 527 rural houses and one rural school across Pampa del Indio municipality along the intervention program. We examined 1,946 out of 9,648 eligible *T. sordida* by OM or vkDNA-PCR collected at 259 rural houses (**Table 1**). Thus, individual examination coverage reached 20%. Considering an insect infected by either vkDNA-PCR or OM, we found a naïve prevalence of *T. cruzi* infection of 1.44% (95% confidence interval [95% CI]: 0.10-2.07%). The prevalence of *T. cruzi* infection detected by vkDNA-PCR (3.42%, n = 527) was significantly higher than the one detected by OM (0.65%, n = 1,529; Fisher’s exact Test p < 0.0001). These naïve prevalences assume 100% sensitivity and 100% specificity. Most of the examined triatomines were collected by timed manual searches (79.1%) or post-timed searches (15.1%), and very few (2.4%) were collected by householders. The sampling fraction of houses with at least one *T. sordida* examined for infection averaged 36.0% (range: 5.6-87.5%) across areas and intervention periods (**Supplementary File 2**). In the last survey, insects were only examined by vkDNA-PCR and the sampling fraction of houses with at least one insect examined for infection was ≥ 87.5% across areas.

**Table 1:** Models of the occurrence and detection of *Trypanosoma cruzi* infection in *Triatoma sordida*, Pampa del Indio, 2007-2009.

Model	AIC	ΔAIC	AIC weight	Model likelihood	K	-2*Log-likelihood
Ψ(adult), p(method)	89.02	0	0.443	1	4	81.02
Ψ (adult+ecotope), p(method)	89.57	0.55	0.336	0.760	5	79.57
Ψ (.), p(method)	92.03	3.01	0.098	0.222	3	86.03
Ψ (ecotope), p(method)	93.63	4.61	0.044	0.100	4	85.63
Ψ (.), p(.)	94.19	5.17	0.033	0.075	2	90.19

The best supported model according to AIC is shown in the first row.

ΔAIC, delta AIC represents the variation of AIC relative to the best model.

k: number of parameters in the model.

Ψ is the occupancy probability (i.e.: probability of *T. cruzi* infection), and p is the detection probability.

We only found one *T. cruzi-*infected specimen of *T. sordida* per house with the exception of three houses (12%) from Area I harboring two infected insects each. Thus, the mean number of infected *T. sordida* per house was 1.12 (SD = 0.33). No house repeatedly harbored infected *T. sordida* among 53 houses where insects were examined for *T. cruzi* infection in at least two different surveys. We examined whether *T. cruzi* infection in *T. sordida* was associated with the occurrence of *T. cruzi* infection in *T. infestans* at the same house and found that 6 (24%) houses harbored both species infected concurrently, 4 of which were detected at baseline, 1 at the early phase and the remaining at the late post-intervention phase. No significant association was found among 120 houses harboring both species and with insects examined for infection (Fisher’s exact test, p = 0.33). Most houses (84%) harboring infected *T. sordida* belonged to Creole families (Fisher’s exact test, p < 0.0001). All houses harboring infected *T. sordida* at baseline had been sprayed with insecticide at the initial attack phase. Houses harboring infected *T. sordida* post-intervention received an average of 0.43 (SD = 0.65) insecticide sprays per house, prompted by the concurrent infestation by *T. infestans*.

### 3.2 Accounting for imperfect detection of *T. cruzi* infection in *T. sordida*

A total of 118 *T. sordida* examined microscopically (8 OM-positive and 110 OM-negative) were subsequently tested by vkDNA-PCR. Most examined triatomines were collected in sites associated with chickens and poultry (72.03%), followed by corrals (20.33%), whereas very few were collected in domiciles (1.69%). Examined insects belonged to 55 houses from Area I; 42.37% of them were collected at baseline and the remaining post-intervention. Co-positivity occurred in 8 (6.78%) insects; co-negativity in 107 (90.68%); and 3 (2.54%) were microscope-negative and vkDNA-PCR-positive.

To estimate detection and infection probabilities we ran five models. The simplest model, considering uniform *T. cruzi* infection probability and uniform detection probabilities across triatomines and techniques, had the least support (**Table 1**). The best model accounted for different detection probabilities for OM and vkDNA-PCR and variation in infection by triatomine stage. The support for the more complex model (in which infection status varied also with collection ecotope) differed little from the best model (ΔAIC < 0.6); therefore, these last two models were considered top models (**Table 1**).

The detection probability of vkDNA-PCR was 100%, SE = 0.00. However, since there was no sample positive for OM and negative for PCR this analyses does not allow an estimate of the true detection probability with certainty. The detection probability for OM was at most 73% (95% CI: 41-91%); hence, considering a detectability of 73% OM missed almost 1 of every 4 infected *T. sordida*. The three OM-false negative outcomes occurred in one male, one female and one fifth-stage nymph. All of them occurred in peridomestic sites associated with chickens from different houses. Infection estimates ranged from 2% to 26% according to developmental stage and collection ecotope, and were higher in adults (versus nymphs) and in triatomines collected in domiciles and other peridomestic sites (versus peridomestic sites associated with chickens). Parameter estimates and 95% CIs are shown in **Table 2**. We have not extrapolated these stage-specific or ecotope-specific infection estimates to the whole study population since this database was intentionally enriched with OM-positive insects.

**Table 2:** Back-transformed numerical estimates from logit to the probability scale.

Model/parameter	Estimate (95% CI)
Ψ(adults), p(method)	
Adults	0.15 (0.08-0.26)
Nymphs	0.03 (0.01-0.13)
OM	0.73 (0.41-0.91)
PCR	1.00 (0.00-1.00)
Ψ(adult+ecotope), p(method)	
Adult + Domiciles and other peridomestic sites	0.26 (0.09-0.54)
Adult + Sites associated with chickens	0.13 (0.06-0.25)
Nymphs + Domiciles and other peridomestic sites	0.05 (0.01-0.21)
Nymphs + Sites associated with chickens	0.02 (0.00-0.11)
OM	0.73 (0.41-0.91)
PCR	1.00 (0.00-1.00)

Ψ is the occupancy probability (i.e.: probability of *T. cruzi* infection), and p is the detection probability.

### 3.3 *Trypanosoma cruzi* infection prevalence adjusted by imperfect detection

Overall, the adjusted *T. cruzi* infection differed significantly with developmental stage (Fisher’s exact test, p = 0.003). It was very low among nymphal stages (range: 0.72-0.88%) and increased among adult insects (**Figure 1**). *Trypanosoma cruzi* infection prevalence among males (3.81%, n = 462) was more than twice as high as among females (1.34%, n = 326)(Fisher’s exact test, p = 0.03), despite similar sampling fractions of insects examined by vkDNA-PCR (38.96%, 95% CI: 34.62-43.48% for males; and 31.60%, 95% CI: 26.78-36.74% for females). Most infected *T. sordida* specimens were collected in peridomestic sites associated with chickens. The prevalence of infection in *T. sordida* peaked in domiciles (4.72%), followed by peridomestic kitchens, storerooms and granaries (3.30%), peridomestic sites associated with chickens (1.4%), corrals (1.42%) and was nil in other peridomestic sites (**Figure 2**) (Fisher’s exact test, p = 0.03). Among householder collections (n = 47), only one *T. sordida* was found infected (**Supplementary File 3**).

**Figure 1:**
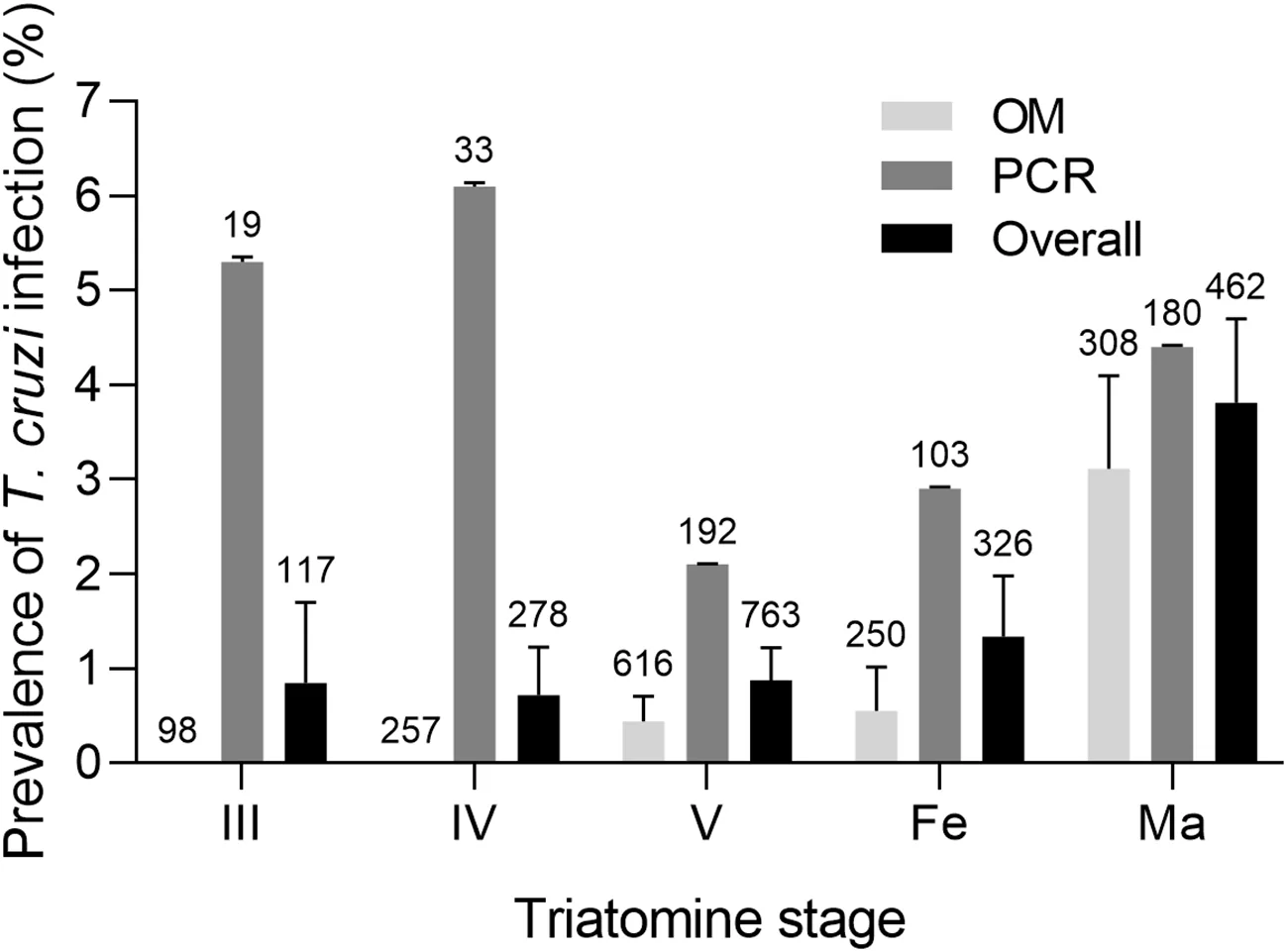
*Trypanosoma cruzi* infection detected by optical microscopy (OM) and vkDNA-PCR (PCR) among *Triatoma sordida* developmental stages accounting for imperfect detection, Pampa del Indio, Chaco; 2007-2016. Numbers above bars represent the number of examined insects; whiskers show the binomial standard error of the prevalence.

**Figure 2:**
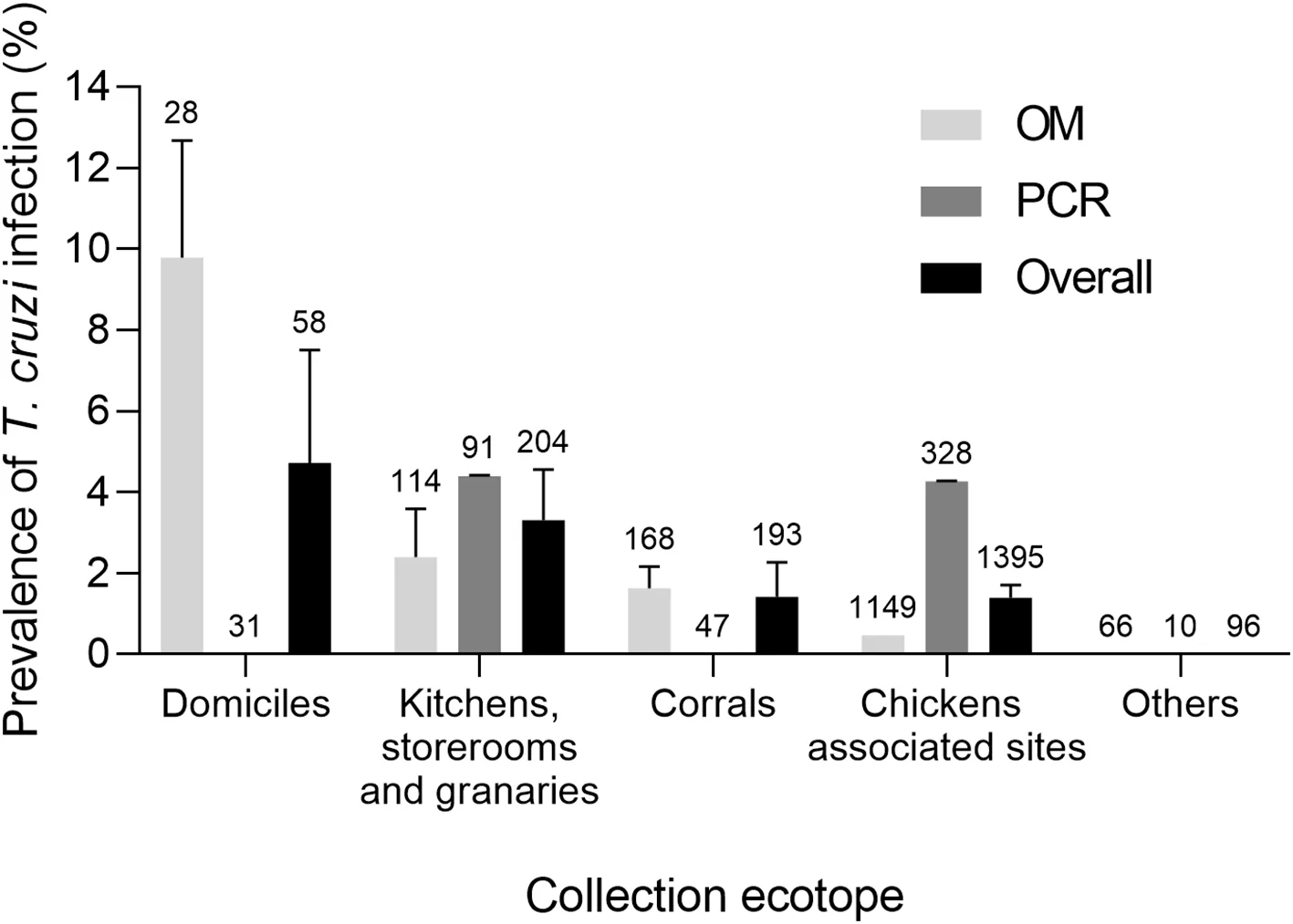
Distribution of *Trypanosoma cruzi* infection in *Triatoma sordida* according to collection ecotopes accounting for imperfect detection, Pampa del Indio, Chaco; 2007-2016. Numbers above bars represent the number of examined insects; whiskers show the binomial standard error of the prevalence.

### 3.4 *Trypanosoma cruzi* infection at the household level

Overall *T. cruzi* infection was detected in a very small proportion (4.73%) of the inhabited houses harboring *T. sordida* and in 9.65% of the houses with examined insects. Most infected *T. sordida* were collected in Area I (85.71%), coincidently with the higher examination effort. However, the overall prevalence of infection was similar among study areas at the insect and household levels (Fisher’s exact tests, p = 0.97 and p = 0.27, respectively) (**Table 3**).

**Table 3:** Distribution of *T. cruzi* infection in *T. sordida* among operational areas, Pampa del Indio, Chaco; 2007-2016.

Operational area	Individual level			Household level		
	N collected*	N examined (%)	% Infected (95% CI)^&^	N infested*	N examined (%)	% infected (95% CI)
Area I	4723	1565 (33.14)	1.77 (1.23-2.58)	242	166 (68.97)	12.65 (8.36-18.63)
Area II	2286	163 (7.13)	1.23 (0.34-4.36)	119	36 (30.77)	5.56 (1.54-18.14)
Area III	998	134 (13.43)	0.75 (0.13-4.11)	62	30 (48.39)	3.33 (0.59-16.67)
Area IV	1641	84 (5.12)	1.19 (0.21-6.44)	99	27 (26.21)	3.70 (0.66-18.28)
Total	9648	1946 (20.17)	1.63 (1.16-2.32)	522	259 (49.62)	9.65 (6.58-13.91)

*Excluding first- or second-instar nymphs and houses only infested with these nymphal stages.

^&^ We have adjusted the infection prevalence according to the OM sensitivity (73%) from occupancy models.

### 3.5 Spatio-temporal distribution of *T. cruzi* infection

The spatial distributions of the occurrence of *T. cruzi* infection at the house level are shown in **Figure 3**. Baseline infection prevalence adjusted by the results of the occupancy model (2.65%, n = 490) was twice as high as in the early (1.26%, n = 1126) and late (1.21%, n = 330) post-intervention phases, though the differences among the three periods were not significant (Fisher’s exact test, p = 0.13) (**Figures 3**, **4**). However, when baseline outcomes were contrasted to pooled post-intervention outcomes, such differences were statistically significant (Fisher’s exact test, p = 0.037). The proportion of insects examined by vkDNA-PCR pre- (28.78%) and post-intervention (26.51%) was similar (Z-test, z-value = 1, p = 0.33). In Area I, *T. sordida* infection declined steadily from 3.37% to 1.45% and nil, at baseline, early and late post-intervention, respectively (Fisher’s exact test, p = 0.01). In other areas, prevalence was nil both at baseline and the early post-intervention phase, and reached 2.74% at the late post-intervention phase (**Figure 4**) (Fisher’s exact test, p = 0.08). However, these differences may be explained by a significantly higher proportion of insects examined by vkDNA-PCR at pre-intervention in Area I and a significantly higher proportion of insects examined by vkDNA-PCR at post-intervention in the other areas (Z-test, z-value = 4.8 and z = 6.3, respectively; p < 0.0001 in both cases).

**Figure 3:**
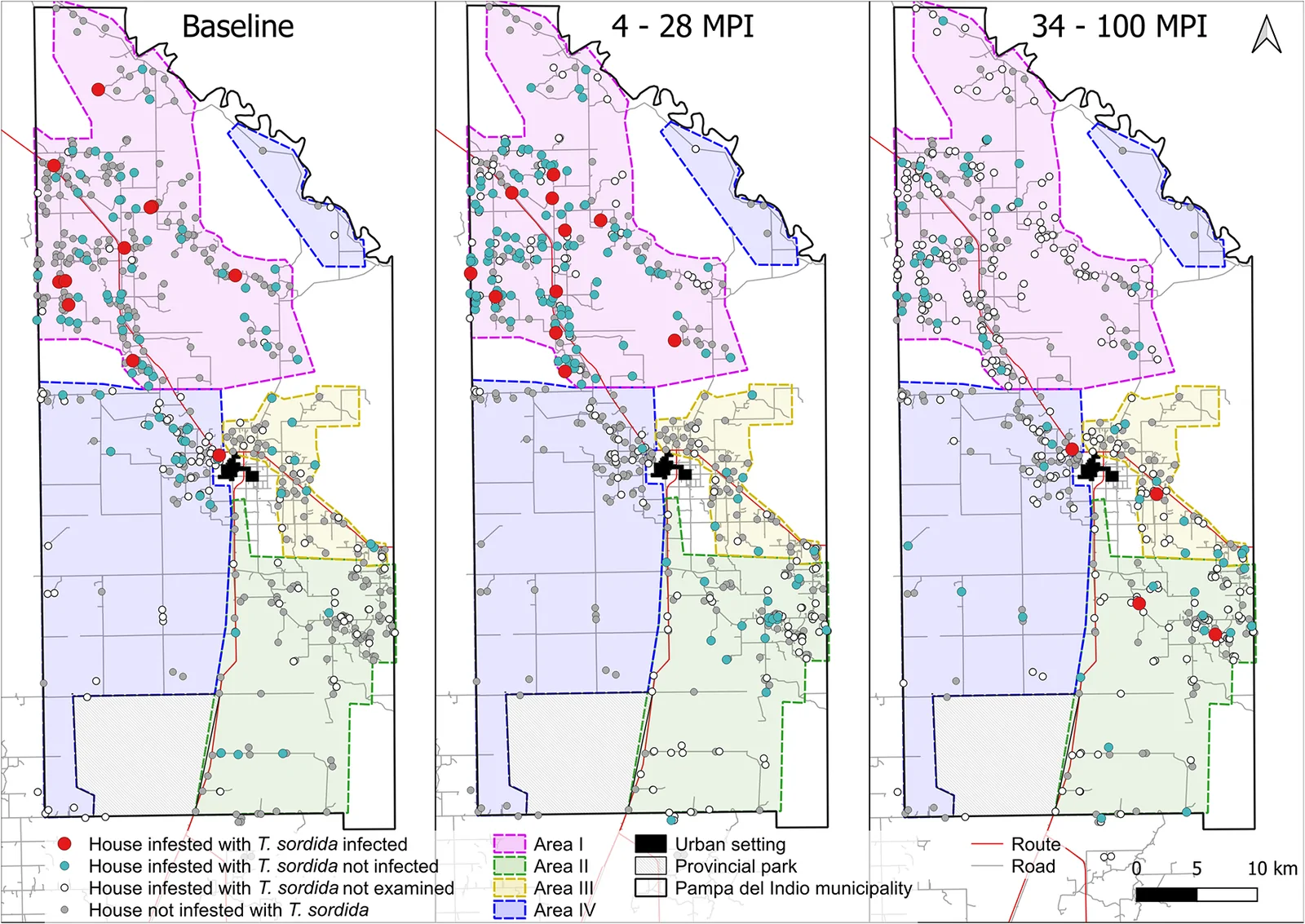
Map showing the occurrence of *Trypanosoma cruzi* infection in *Triatoma sordida* according to intervention period in Pampa del Indio, 2007–2016. a: Baseline, 0 MPI; b: Early, 4–28 MPI, and c: Late post-intervention phase, 34–100 MPI. Each dot represents a rural house, grey dots: houses not infested with *Triatoma sordida;* white dots, houses harboring *T. sordida* that were not examined for infection. Cyan and red dots, houses harboring *T. cruzi* -infected or -uninfected *T. sordida*, respectively.

**Figure 4:**
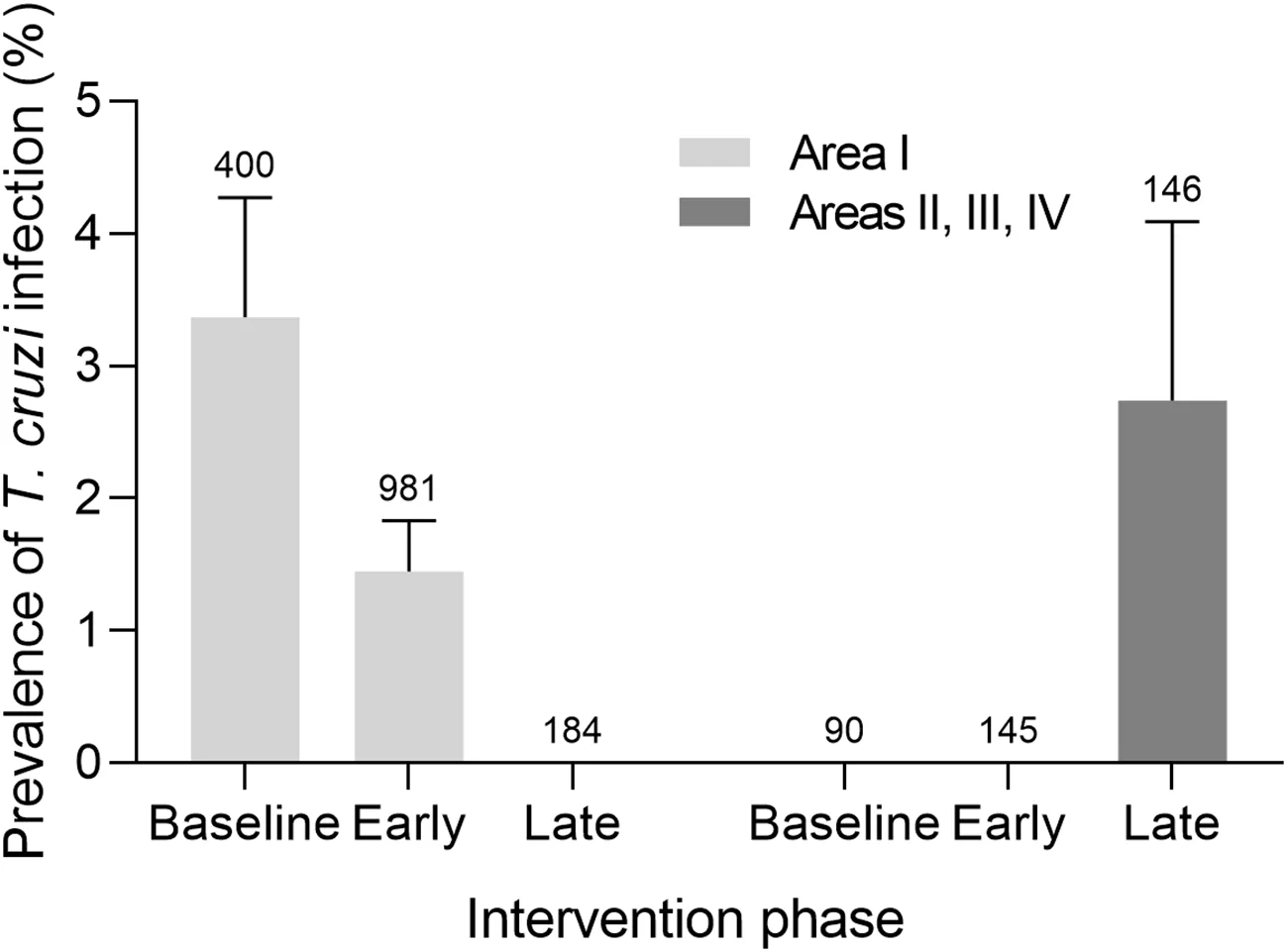
Distribution of *Trypanosoma cruzi* infection in *Triatoma sordida* according to operational area and intervention period in Pampa del Indio 2007–2016. Baseline, 0 months post-intervention [MPI]; Early post-intervention phase, 4–28 MPI, Late post-intervention phase, 34–100 MPI. Numbers near bars represent the number of examined insects; whiskers show binomial standard errors.

No significant spatial pattern of *T. cruzi* infection in *T. sordida* was detected via global spatial analysis (**Supplementary File 4**). Local spatial analysis revealed one significant hotspot of *T. cruzi* infection in *T. sordida*, located in the northwestern section of the municipality (Area I) (**Figure 5**). This hotspot exhibited a significant aggregation with a minimum radius of 200 m and a maximum of 1,300 m and encompassed 24 houses, 7 of them harboring infected *T. sordida*.

**Figure 5:**
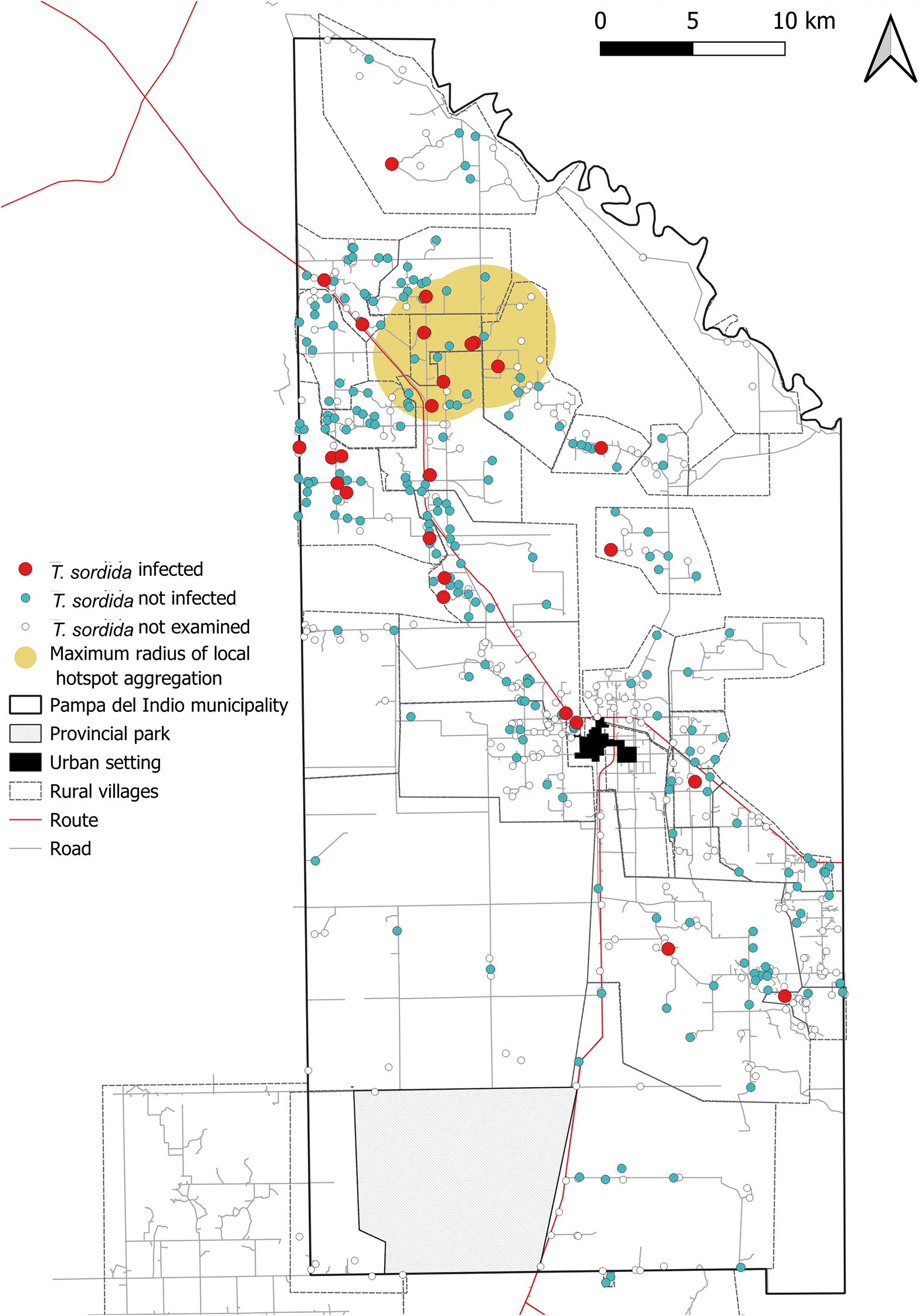
Hotspot of houses with *T. cruzi*-infected *T. sordida* along 2007-2016, Pampa del Indio, Chaco.

### 3.6 DTU identification

We achieved a 150-157bp-band in the UTCC-TcAc PCR in five infected specimens of *T. sordida* in this study, and no bands in the other PCRs despite several attempts to identify parasite DTUs. Hence, infections by TcIII or TcIV could be discarded but no conclusive DTU identification among TcI, TcII, TcV or TcVI was achieved. PCR inhibitors were ruled out as the cause of no amplification since triatomines subjected to DTU identification have all resulted vkDNA-PCR positive. Along the intervention, TcI predominated in all the infected *T. sordida* detected as reported previously (Maffey et al., 2012; Macchiaverna et al., 2015; Provecho et al., 2014) (**Supplementary File 3**).

## 4 Discussion

Our results show a relatively low prevalence of *T. cruzi* infection in *T. sordida* both at pre- and post-intervention (2007-2016), even after accounting for imperfect detection of infection. The declining infection prevalence in *T. sordida* detected in parallel to the quasi-elimination of *T. infestans* from rural houses contrasts with findings from elsewhere in the Gran Chaco where *T. sordida* gained epidemiological importance and calls for new studies to explain these patterns like bloodmeal identification and triatomine species determination using molecular techniques. We also report the occurrence of an infection hotspot where infection was concentrated at baseline and the early post-intervention period. The use of vkDNA-PCR significantly improved the detectability of *T. cruzi* infection in this species. The estimated detection probability of OM for *T. sordida* specimens and *T. infestans* (Cardinal et al., 2025) were very similar (73% and 76%, respectively).

*Triatoma sordida* was the most frequently species captured in rural houses of Pampa del Indio municipality since the first year post-intervention. This predominance has also been observed in Brazil, Paraguay and Bolivia, though in association with different levels of *T. cruzi* infection. In these countries and in Colombia, after eliminating the main domestic vectors, native species of triatomines exhibited high levels of *T. cruzi* infection and jeopardized the control of transmission achieved (Bezerra et al., 2018; Cantillo-Barraza et al., 2021; Gonzalez-Britez et al., 2021; Guilherme et al., 2001; Sánchez et al., 2020). Conversely, in Pampa del Indio, over the nearly 10 years of vector surveillance and control, *T. cruzi-*infected *T. sordida* remained at very low levels (<2% of the examined insects, ≤5% of inhabited houses harboring *T. sordida*). In Santiago del Estero, in the dry Argentine Chaco, Cecere et al. (1999) reported a prevalence of 0.2% among 832 *T. sordida* specimens examined microscopically. No infected *T. sordida* (or other secondary species) were reported across Argentina (Bar et al., 1993; Cardozo et al., 2021; Marti et al., 2009), though triatomine samples sizes were small (< 100).

Most *T. cruzi*-infected *T. sordida* specimens were collected in peridomestic sites associated with chickens and other poultry, and both turkeys and chickens were bloodmeal sources of the infected *T. sordida* (Macchiaverna et al., 2015). Infected *T. sordida* were also frequently collected in chicken coops in Mina Gerais, Brazil (Valença-Barbosa et al., 2025). The finding of infected insects in ecotopes harboring avian hosts may be explained by the outstanding dispersal ability (Forattini et al., 2006, 1983) and opportunistic feeding capacity of *T. sordida* (Pires et al., 1999).

*Trypanosoma cruzi* exhibits a high genetic diversity and seven DTUs (i.e.: genotypes) have been described so far (Zingales et al., 2012, 2018). DTUs display differential distributions among geographical areas, hosts and transmission cycles. In Argentina, TcII, TcV and TcVI predominate in the domestic transmission cycles (Cardinal et al., 2008; Enriquez et al., 2013; Macchiaverna et al., 2018; Maffey et al., 2012; Monje-Rumi et al., 2013) whereas TcIII and TcI predominate in the sylvatic one (Alvarado-Otegui et al., 2012; Ceballos et al., 2006; Diosque et al., 2003; Enriquez et al., 2013; Macchiaverna et al., 2015; Orozco et al., 2013). The most frequent DTU identified in the infected *T. sordida* from Brazil was TcI, though TcII, TcIII and TcV were also reported (Valença-Barbosa et al., 2025). A predominance of TcI was also recorded in Pampa del Indio. Overall, TcI predominated (67%) over hybrid lineages (TcV, TcVI and TcV/TcVI) (Maffey et al., 2012; Macchiaverna et al., 2015). Hybrid lineages were mainly collected in domiciles and peridomestic kitchen, corral and granary, whereas TcI were mainly collected in chicken-associated peridomestic structures. All of these DTUs were identified at baseline and at the early post-intervention. TcI was also detected in a *T. sordida* collected in a peri-urban house (Provecho et al., 2014). TcI infecting *T. sordida* were identified as TcId and clustered with other TcI parasites detected in *Didelphis albiventris* opossums (Macchiaverna et al., 2025), pointing to the latter as the potential source of *T. cruzi* in peridomestic *T. sordida*, highlighting its potential role as a bridge vector between sylvatic and peridomestic transmission cycles.

We found that adult *T. sordida* exhibited a higher prevalence of infection than nymphs, a pattern widely observed in *T. infestans* (e.g. Gürtler et al., 2023a) driven by cumulative exposure to potentially infectious bloodmeals across the lifespan and increasing bloodmeal size with stage. However, the higher *T. cruzi* infection detected in *T. sordida* males is hard to explain, since *T. sordida* males exhibited a shorter lifespan than females in a laboratory setting (Pacheco de Souza et al., 1978). No sex-specific differences in the susceptibility to *T. cruzi* infection have been reported among different triatomine species including *T. sordida* (Phillips and Bertram, 1967). There is no clear evidence whether males disperse more frequently than females by flight in triatomines and whether this behavior implies a higher infection probability. A few studies reported a higher number of males of *T. garciabesi* or *T. sordida* dispersing by flight, though numbers were relatively small (Di Iorio and Gürtler, 2017; Noireau and Dujardin, 2001; Vazquez-Prokopec et al., 2004, 2006). If householder collections are taken as an index of invasive triatomine behavior, in this study, householders collected nearly twice as many female *T. sordida* as males, in accordance with experimental data showing that female *T. sordida* engaged in more flights than males (Firmino et al., 2025). Potential drivers of the higher infection in males include more frequent bloodmeals, greater movement and exposure to a greater diversity of hosts; as speculated previously for other triatomines species (Curtis-Robles et al., 2018). More research is needed to elucidate whether there are sex differences in infection prevalence and the mechanisms involved.

A strikingly different spatial pattern of houses with *T. cruzi-*infected *T. sordida* was observed at the late post-intervention phase rather than at previous phases. At baseline and early post-intervention, infection was concentrated at the north-western section of the municipality (Area I), whereas in the late phase, the few infected specimens were collected in the central-eastern section of the municipality across areas II-IV (**Figure 3**); coincidently with a decrease of *T. cruzi* infection in the *T. sordida* collected in Area I. This could be explained if infection in *T. sordida* at baseline and early post-intervention originated from both the domestic and sylvatic transmission cycles and later on, when domestic transmission mediated by *T. infestans* was virtually suppressed, *T. cruzi* infection in *T. sordida* only originated from the sylvatic cycle. DTUs identification gives support to this hypothesis; TcVI and hybrid lineages (DTUs associated with the domestic transmission cycle in the Argentinean Chaco) were only detected in *T. sordida* at baseline and the early post-intervention whereas TcI infections were detected along the whole intervention. Molecular epidemiology linked the TcI parasites infecting *T. sordida* with the TcI infecting *Didelphis albiventris* opossums in Pampa del Indio (Macchiaverna et al., 2025).

Ìnfected *Didelphis albiventris* opossums were caught across Area I between 2008 and 2011 (Orozco et al., 2013) close to the *T. sordida* infection hotspot. If patches of certain vegetation types could favor the co-ocurrence of sylvatic *T. sordida* and opossum populations, the observed hotspot may be linked to these vegetation patches, which remained to be determined. Other underlying social or ecological drivers of infection hotspot in Area I merit further research. Insecticide treatments targeting *T. infestans* were followed by short-term reductions in the abundance of *T. sordida* with subsequent fast recovery and marked seasonal fluctuations in Area I (Rodríguez-Planes et al., 2020). We enquired whether fewer insecticide applications might explain the observed spatial distribution of infected *T. sordida* post-intervention. The 14 houses harboring infected *T. sordida* post-intervention received more (mean = 0.48, SD: 0.65) insecticide sprays than the rest of rural houses across the municipality (mean = 0.13, SD: 0.44) (Gürtler et al., 2023a). Alternatively, the relatively higher infection prevalence detected in Areas II, III and IV post-intervention could be due to unbalanced proportions of insects examined by vkDNA-PCR.

Intense deforestation and land use change occurred along the intervention program. Disentangling how these two anthropogenic changes potentially affected sylvatic *T. sordida* populations, dispersal dynamics and house invasion and colonization is beyond the scope of the present study.

Our study has some strengths and limitations. We have consistently utilized the same protocols for triatomine infestation assessment and parasitological diagnosis along the intervention program, which enabled us to compare current results with previous findings. Accounting for imperfect detection improved the accuracy of infection estimates. The variable proportion of examined insects and households across areas and periods may have introduced some bias given that most of the *T. sordida* specimens originated from Area I and the proportion of examined insects by vkDNA-PCR differed among areas. Nevertheless, in our last timepoint, all insects were examined by vkDNA-PCR across study areas. The small number of houses harboring infected *T. sordida* impeded further analysis of the co-occurrence of infection in both *T. infestans* and *T. sordida.* Lack of molecular confirmation for *T. sordida* subcomplex species implies that more than one species may have been collected. Large differences among domestic or peridomestic ecotopes in terms of hosts availability and microhabitat stability give further support to this possibility. In addition, the Chaco province overlaps with the potential geographical distribution of three *T. sordida* clades (Burgueño-Rodríguez et al., 2025). However, a recent study showed that *T. sordida* sensu stricto, *T. rosai* and their hybrids exhibited similar feeding and defecation patterns (Vicente et al., 2022). Thus, working with *T. sordida* sensu lato seems to have no clear implication on the vector competence. We also were unable to identify the infecting DTU in *T. sordida* detected in this study, in contrast with those collected at baseline and the early post-intervention phase. Whether this was due to the low parasite burdens in *T. sordida* (Valença-Barbosa et al., 2025) remains unknown.

In parallel to the ecological processes of domiciliation and adaptation to human-modified environments, secondary triatomine vectors display a variety of traits related to vectorial capacity. Although we have found low levels of *T. cruzi* infection in house populations of *T. sordida* at a district-wide scale over nearly a decade, infection prevalence is only one component of transmission risk. Given the enzootic nature of *T. cruzi* transmission cycles, our imperfect knowledge on how they function, and the finding of infected *T. sordida* in the late post-intervention phase, sustained surveillance within an adaptive management scheme is needed for long-term interruption of vector-borne transmission.

## Data Availability

The original contributions presented in the study are included in the article/**Supplementary Material**. Further inquiries can be directed to the corresponding author/s.
